# Contribution of the Skin–Gut Axis to Immune-Related Adverse Events with Multi-System Involvement

**DOI:** 10.3390/cancers14122995

**Published:** 2022-06-17

**Authors:** Alyce M. Kuo, Lukas Kraehenbuehl, Stephanie King, Donald Y. M. Leung, Elena Goleva, Andrea P. Moy, Mario E. Lacouture, Neil J. Shah, David M. Faleck

**Affiliations:** 1 Dermatology Service, Department of Medicine, Memorial Sloan Kettering Cancer Center, New York, NY 10021, USA; alyce.kuo@icahn.mssm.edu (A.M.K.); lacoutum@mskcc.org (M.E.L.); 2 Ludwig Collaborative and Swim Across America Laboratory, Parker Institute for Cancer Immunotherapy, Human Oncology and Pathogenesis Program, Memorial Sloan Kettering Cancer Center, New York, NY 10065, USA; 3 Gastroenterology, Hepatology & Nutrition Service, Department of Medicine, Memorial Sloan Kettering Cancer Center, New York, NY 10065, USA; kings2@mskcc.org (S.K.); faleckd@mskcc.org (D.M.F.); 4 Division of Allergy-Immunology, Department of Pediatrics, National Jewish Health Hospital, Denver, CO 80206, USA; leungd@njhealth.org (D.Y.M.L.); golevae@njhealth.org (E.G.); 5 Dermatopathology Service, Department of Pathology, Memorial Sloan Kettering Cancer Center, New York, NY 10065, USA; moya@mskcc.org; 6 Genitourinary Solid Tumor Service, Department of Medicine, Memorial Sloan Kettering Cancer Center, New York, NY 10065, USA; shahn6@mskcc.org

**Keywords:** immune checkpoint blockade, cutaneous adverse events, rash, bullous pemphigoid, diarrhea, colitis

## Abstract

**Simple Summary:**

Increasing numbers of cancer patients are treated with immunotherapy that activates their immune systems to control or even eliminate tumors. However, a substantial proportion of patients experience adverse events mediated by the unleashed immune system. The skin is one of the most frequently affected organs, with toxicities typically manifesting as distinct types of rashes. The gastrointestinal (GI) tract is also commonly affected, with a wide spectrum of symptom manifestations that can range from self-limited diarrhea to life-threatening colitis. Here we present the relationship between skin and GI adverse events among cancer patients receiving treatment with immune checkpoint blockade, which has not been well-studied.

**Abstract:**

Immune-related adverse events (irAEs) frequently complicate treatment with immune checkpoint blockade (ICB) targeting CTLA-4, PD-1, and PD-L1, which are commonly used to treat solid and hematologic malignancies. The skin and gastrointestinal (GI) tract are most frequently affected by irAEs. While extensive efforts to further characterize organ-specific adverse events have contributed to the understanding and management of individual toxicities, investigations into the relationship between multi-organ toxicities have been limited. Therefore, we aimed to conduct a characterization of irAEs occurring in both the skin and gut. A retrospective analysis of two cohorts of patients treated with ICB at Memorial Sloan Kettering Cancer Center was conducted, including a cohort of patients with cutaneous irAEs (ircAEs) confirmed by dermatologists (*n* = 152) and a cohort of patients with biopsy-proven immune-related colitis (*n* = 246). Among both cohorts, 15% (61/398) of patients developed both skin and GI irAEs, of which 72% (44/61) patients had ircAEs preceding GI irAEs (*p* = 0.00013). Our study suggests that in the subset of patients who develop both ircAEs and GI irAEs, ircAEs are likely to occur first. Further prospective studies with larger sample sizes are needed to validate our findings, to assess the overall incidence of co-incident irAEs, and to determine whether ircAEs are predictors of other irAEs. This analysis highlights the development of multi-system dermatologic and gastrointestinal irAEs and underscores the importance of oncologists, gastroenterologists, and dermatologists confronted with an ircAE to remain alert for additional irAEs.

## 1. Introduction

Immune checkpoint blockade (ICB) generates anti-tumor response through the inhibition of cytotoxic T-lymphocyte-associated protein 4 (CTLA-4), programmed cell death protein 1 (PD-1), and/or programmed cell death protein 1 ligand (PD-L1). Tumor cells engage these targets to promote quiescence of T-cells, and inhibition of these targets allows for the activation of T-cells against tumor cells [1]. Although ICB is now widely used and improves survival in a multitude of malignancies, including melanoma and other skin, lung, and genitourinary cancers [2,3,4], the development of immune-related adverse events (irAEs) negatively impacts quality of life and frequently leads to treatment interruptions. Various organ systems can be affected by irAEs, though the skin, gastrointestinal, endocrine, and pulmonary systems are affected most often. It is unclear why some patients are more affected by irAEs than others, although some evidence suggests that irAEs may be associated with increased ICB efficacy and patient survival [5,6,7].

Immune-related cutaneous adverse events (ircAEs) occur early during ICB; for instance, maculopapular rashes may present within 6 weeks of the initial ICB dose. Other ircAEs which have been associated with ICB include lichenoid eruptions, psoriasiform rashes, and immunobullous reactions [8,9]. IrcAEs are also the most frequent irAEs, affecting up to 30–60% of patients on ICB [10]. Most ircAEs are mild to moderate (grades 1–2 per the Common Terminology Criteria for Adverse Events (CTCAE) version 5.0), with severe toxicities (grade 3 or 4) observed in <20% and <25% of patients on anti-PD1/PDL1 and anti-CTLA4 therapies, respectively [10]. However, the impact on a patient’s quality of life can be profound [11,12,13].

Similarly, gastrointestinal irAEs (GI irAEs) also affect patients’ quality of life. They can be life-threatening and represent the most common severe (grades 3–4) irAEs. Additionally, GI irAEs account for the leading cause of treatment discontinuation [14,15,16]. GI irAEs typically occur later than cutaneous toxicities, with a median onset of 6-8 weeks, and most frequently present as immune-related colitis (irColitis) and diarrhea [14,17]. Mild to moderate (CTCAE grade 1 or grade 2) diarrhea includes an increase of up to 6 stools per day and grade 2 colitis presents with abdominal pain, or mucus or blood in the stool. Grade 3 diarrhea is defined as an increase in 7 or more stools per day and grade 3 colitis can present with severe abdominal pain, fever, and abdominal signs [18]. As ircAEs and GI irAEs are among the most frequently observed irAEs, these events may co-occur in many patients. However, the relationship between ircAEs and GI irAEs, and the frequency of patients developing both is not well-characterized.

## 2. Patients and Methods

We performed an IRB-approved (IRB protocol #16-458) retrospective analysis of two patient cohorts.

To identify cases of ircAE, a systematic search of electronic medical records was conducted with the help of MSK’s DataLine team. Patients treated with ICB between September 2017 and September 2019 were identified, and patients with a SEER diagnosis for hematologic malignancy were excluded. This search identified 3569 individual patients. To identify potential cases of ircAE, we further searched for ICD-10 codes potentially associated with an ircAE. Thereby, 398 suspected cases of ircAE were identified that subsequently underwent manual chart review by a dermatologist including clinical documentation, photographs, and histopathology (where available). This review confirmed 152 cases of ircAE (Cohort 1). Search criteria are detailed in Table S1. The medical records of patients in Cohort 1 were subsequently assessed for evidence of GI irAE development (diarrhea, abdominal pain, etc., attributed to ICB use).

Cohort 2 was comprised of patients treated with ICB between January 2006–July 2021 who were referred to gastroenterology and subsequently had lower endoscopy (flexible sigmoidoscopy or colonoscopy) with biopsy results reviewed by expert GI pathologists consistent with irColitis. Medical records of patients in Cohort 2 with biopsy-confirmed irColitis and a documented dermatology visit were evaluated for evidence of ircAE development (rashes, pruritus, and other skin changes attributed to ICB use).

Descriptive statistics were used to summarize the patient demographics and clinical characteristics. The two-sample t-test with equal variance was utilized to detect differences in age between Cohort 1 and Cohort 2. The chi-squared test was used to compare categorical variables between Cohort 1 and Cohort 2 (sex, race, ethnicity, ICB type, and cancer type) and to compare cancer types, and ircAE or irColitis occurrences. Binomial probability was assessed for ircAE preceding GI irAE using the Bernoulli model, and cumulative probability is reported. Analyses were considered significant if *p* < 0.05 and there was no adjustment for multiplicity. All analyses were performed using Stata, version 16 (StataCorp LLC, College Station, TX, USA).

## 3. Results

In Cohort 1, 152 patients were identified and confirmed to have developed ircAEs secondary to ICB. In Cohort 2, 246 patients had biopsy-confirmed irColitis. The most commonly treated cancers were genitourinary malignancies in Cohort 1 and melanoma in Cohort 2 (Table 1). In both, pembrolizumab was the most common ICB associated with irAEs (Table 1).

### 3.1. Cohort 1

**Figure 1:**
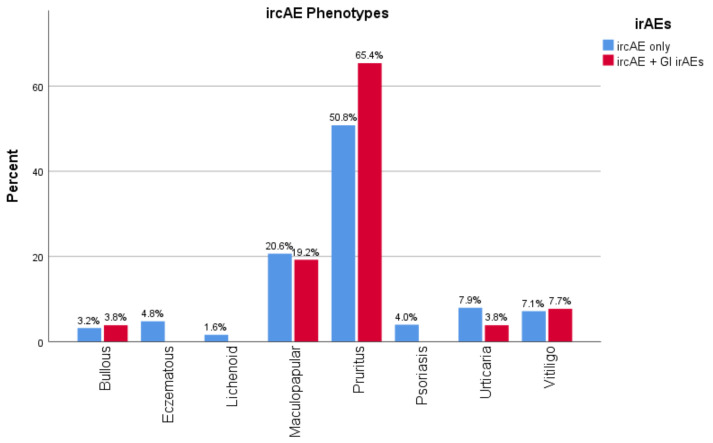
ircAE phenotype and GI irAE development.

Among those who had verified ircAEs in Cohort 1, 17.1% of patients (26/152) also experienced diarrhea (Table 2). IrcAE phenotypes were relatively similar among patients who developed only ircAEs, and those who developed both ircAEs and GI irAEs. Although patients with the pruritus ircAE phenotype (defined as pruritus without apparent skin changes other than excoriations/scratch marks) more often also experienced GI irAEs; this trend was not significant (Figure 1). Of the patients who experienced both ircAE and GI irAE, 69.2% (18/26) developed ircAE before GI irAE. No significant association between ICB target and effect on both skin and gut was identified (Table 2).

A subset-analysis was conducted for lung cancer, melanoma, GU, and other. No significant differences were found, and the main trend of ircAE preceding GI irAE was maintained throughout all cancer types, with the exception of lung cancer, where three patients each had ircAE leading to GI irAE and GI irAE preceding ircAE (Table S2).

**Figure 2:**
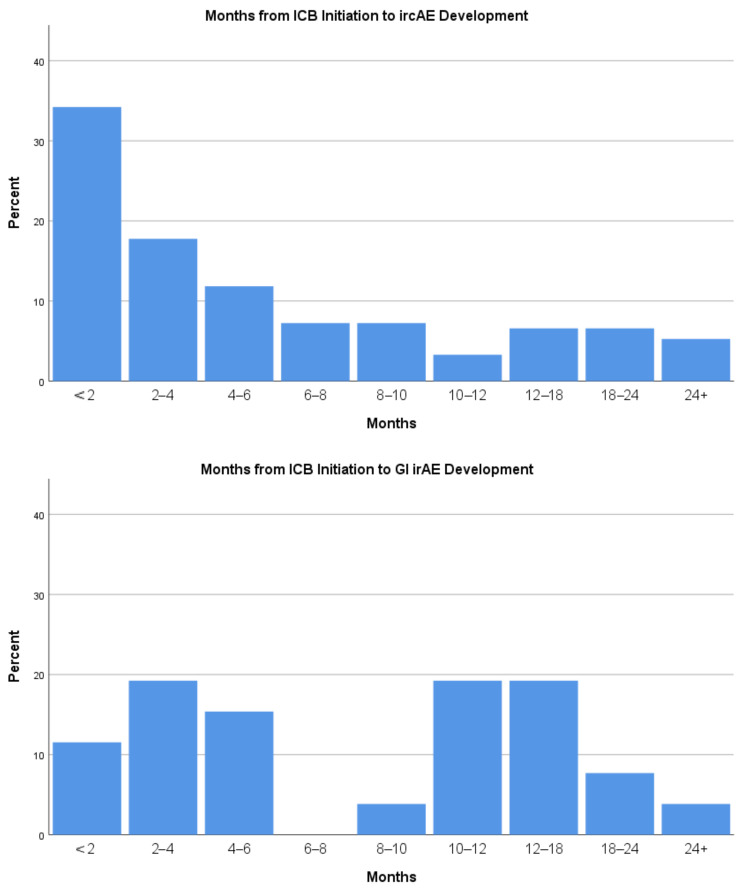
Months from ICB initiation to ircAE and GI irAE development.

While the majority of ircAEs in this cohort developed within 6 months of ICB initiation, GI irAEs were observed more than 6 months and even more than a year after ICB initiation (Figure 2).

Patients on all ICB regimens developed both ircAEs and GI irAEs, with no statistically significant association between regimens including anti-CTLA4 blockade vs. those without, and the development of both ircAEs and GI irAEs (*p* = 0.456).

### 3.2. Cohort 2

**Figure 3:**
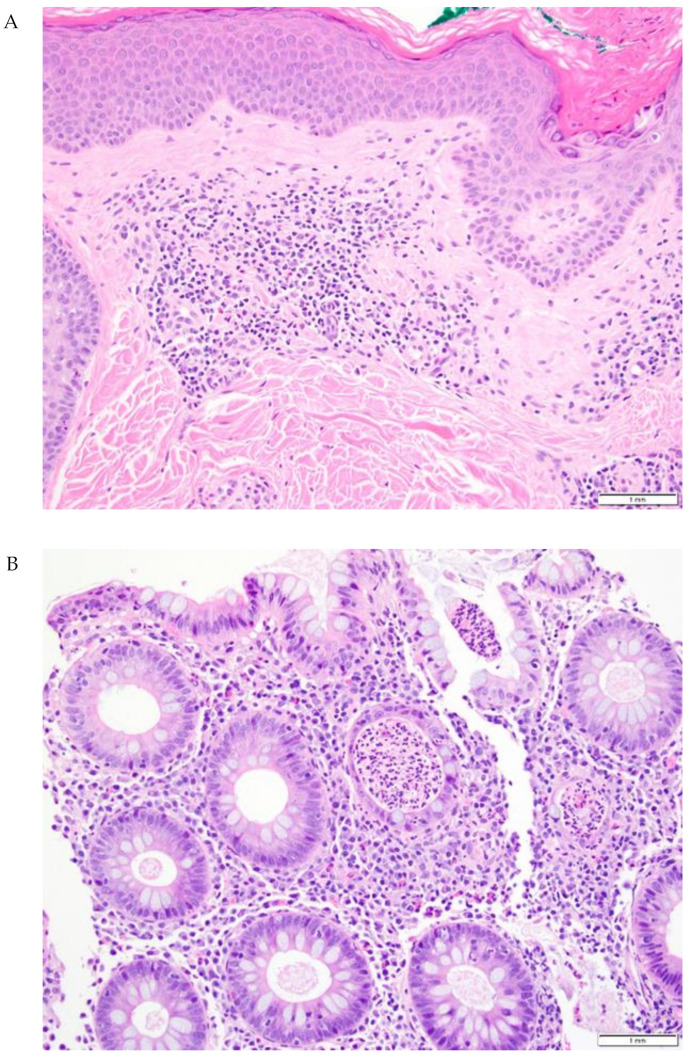
Representative skin and gut biopsies (20×) from a patient with both ircAE and irColitis. (**A**) Lichenoid dermatitis with eosinophils and focal acantholytic dermatosis. (**B**) Active colitis with crypt abscesses, scattered intraepithelial lymphocytes, and increased inflammatory cells in lamina propria.

Two hundred and forty-six patients had lower endoscopy and biopsy-confirmed irColitis. Of these, 14.2% (35/246) also experienced ircAE (Figure 3). Notably, 74.3% (26/35) developed ircAE before irColitis (Table 3). In patients who had both irColitis and ircAE, maculopapular rash was the most common ircAE phenotype. Furthermore, an association between combination treatment targeting both CTLA4 and PD1/PD-L1 was associated with developing ircAE in addition to GI irAE (odds ratio 10.623 for combination vs CTLA4 monotherapy, *p* = 0.02).

In a subset analysis by cancer type, a statistically significant difference was found with frequencies of multiorgan involvement. Notably, in lung cancer, only 2 out of 46 patients (4%) also developed ircAE (*p* = 0.019). However, the trend of skin preceding gut was maintained in all sub-cohorts (Table S2).

### 3.3. Patients with Both ircAEs and GI irAEs

**Figure 4:**
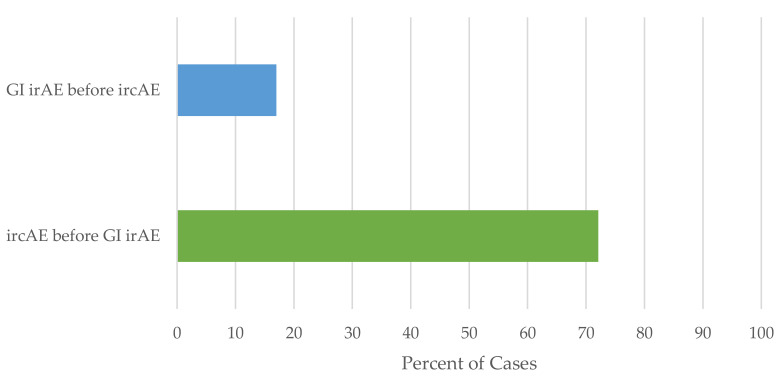
Relationship between ircAEs and GI irAEs.

Across both cohorts, in the 61 patients who developed both ircAEs and GI irAEs associated with ICB, ircAEs occurred before GI irAEs in 72.1% (44/61) of patients (Figure 4). Using the Bernoulli model, the likelihood of this occurring at random is 0.00013.

## 4. Discussion

We sought to evaluate two large patient cohorts: the first cohort with ircAEs verified by a dermatologist, and the second cohort with lower endoscopy and biopsy-confirmed irColitis to ensure inclusion of patients with confirmed GI irAEs as assessed by an expert in oncological gastroenterology. Our analysis shows that 10–20% of patients on ICB develop both ircAEs and GI irAEs over the course of ICB treatment. Interestingly, although anti-CTLA4-based regimens are historically associated with higher rates of GI irAEs, we find that in patients who also develop ircAEs, proportions of GI irAEs are similar between anti-PD1- or anti-CTLA4-based regimens. This could be explained by a corresponding higher incidence of ircAEs in anti-CTLA4-based regimens as well.

In patients who developed both skin and gut irAEs, we observed that ircAEs frequently occurred first. This is consistent with prior reports that ircAEs tend to develop earlier than toxicities affecting other systems [19,20]. While previous reports have associated mucositis with increased risk of GI irAE development [20], patients in our cohorts with all ircAE phenotypes developed GI irAEs. This underscores the importance of monitoring all patients who develop ircAEs of any phenotype for any symptoms of GI irAEs, such as diarrhea. Conversely, patients who develop GI irAEs should be counseled about the possibility of ircAE development. Earlier recognition of irAE development and swift management may allow patients to maintain ICB treatment, which might further improve the outcome of patients who develop irAEs [21].

When analyzing the most frequent cancer types in this study separately, the finding of ircAE preceding GI irAE is maintained. Overall, this sub-analysis needs to be interpreted cautiously due to the small sample size. Frequencies of multiorgan irAE are also comparable to the overall results. However, an intriguing difference is found in Cohort 2, where only 4% of lung cancer patients develop ircAE in addition to irColitis. The differences by cancer type in this cohort are statistically significant and warrant further investigation. Whilst ircAEs are generally reported to be the most frequent irAEs, in a trial of first-line nivolumab for NSCLC, GI irAE were reported slightly more frequently than ircAE [22], ircAEs were the most frequently reported irAE in a study of pembrolizumab for NSCLC [23], and comparable frequencies of GI irAE and ircAE were reported in a combination of ipilimumab and nivolumab for NSCLC [24]. A slight trend towards lower relative incidence of ircAE in lung cancer patients is therefore conceivable but unlikely to wholly account for the finding. Considering that cohort 2 consists of patients with biopsy-proven irColitis that have been referred to dermatology, a referral bias is a potential explanation, i.e., milder dermatologic toxicities may not have been captured and/or referred. Thoracic oncologists might manage more ircAE independently, perhaps due to longer experience as this was the second FDA approved cancer to be treated with ICB after melanoma. This observation warrants further investigation in subsequent retrospective- or preferably prospective studies.

In cohort 2, consisting of patients with biopsy-confirmed irColitis, PD-1 blockade in addition to CTLA-4 blockade increased the risk of additional skin toxicity. This observation might emphasize that CTLA-4 blockade preferentially leads to GI irAE. 

Although the underlying pathogenesis of irAEs in various organ systems has not been clearly elucidated, many hypotheses have been posed. T-regs that express CTLA-4 are essential to maintaining homeostasis in the gut by promoting the suppression of effector T-cell activity and by increasing peripheral tolerance [25]. Notably, *CTLA-4* gene polymorphisms have been associated with irritable bowel disease [26,27]. Therefore, it is conceivable that anti-CTLA-4 therapy could lead to gut dysregulation and GI irAEs [28]. Additionally, the inhibition of CTLA-4 expressing T-regs may lead to the overactivation of Th1 and Th17 axes [29]. Likewise, blocking the PD-1 pathway with anti-PD-1/PD-L1 therapy may result in the proliferation of self-reactive T-cells [30]. It has been noted that the frequency of PD-1 expressing CD8 cells relative to PD-1 expressing T-regs can predict the clinical efficiency of PD-1 blockade therapies, suggesting that the variability in PD-1 expression on regulatory and effector T-cells may influence the development of irAEs while on ICB [31]. Immune activation of T-cells in Peyer patches has also been associated with the induction of CLA+ T-cells, which are known to be skin homing and therefore may create a conduit between the gut and skin.

The skin and gut share many characteristics and functions, including serving as epithelial bordering organs with comparable antimicrobial proteins and defense roles [32], and these similarities may contribute to the development of both ircAEs and GI irAEs in patients treated with ICB. Many dermatologic conditions have gastrointestinal manifestations, and many gastrointestinal disorders have cutaneous involvement as well. For example, Stevens—Johnson syndrome involves sloughing of large body areas of skin and at least two mucous membranes. Erythema, edema, and friability can develop in the gastrointestinal tract as well. In inflammatory bowel disease, the skin can be involved with pyoderma gangrenosum and/or erythema nodosum. Hereditary disorders may affect both the skin and the gut as well, such as in Muir—Torre syndrome, in which affected patients can present with sebaceous adenomas and develop multiple colon polyps [33].

Furthermore, the concept of a skin—gut axis (also known as the gut—skin axis or gut—skin—brain axis) is increasingly described, particularly in the setting of inflammatory diseases [34,35,36]. Emerging evidence suggests that there may be not only a close relationship but also signaling and communication between the skin and the gut, so that disruption in one then leads to disturbances in the other as well. Specifically, it is thought that microbiota in the gut, including *Bacteroides fragilis* and *Faecalibacterium prausnitzii*, stimulate Treg and lymphocyte proliferation and promote anti-inflammatory responses systemically by producing short chain fatty acids through fermentation. Short chain fatty acids play a role in the inhibition of inflammatory cell proliferation, migration, adhesion, and cytokine production [34]. In addition, intestinal microbiome production of inosine has been shown to enhance ICB efficacy through adenosine A2 co-stimulation of effector T-cells [37]. Metabolites from the gut microbiota can also migrate to the skin to upset cutaneous homeostasis [34]. Dysbiosis in the gut has been associated with the development of cutaneous disorders such as acne vulgaris, atopic dermatitis, psoriasis, and rosacea [36]. Similarly, metabolites from skin inflammation may disturb the gut as well. New evidence shows that inflammation in the skin leads to the catabolism of hyaluronic acid, releasing fragments that can trigger the differentiation of intestinal fibroblasts into preadipocytes. Preadipocytes have innate immune function, and therefore, this signaling would, in theory, prime the gut for the source of skin inflammation. However, it also results in reactive adipogenesis, which is recognized in inflammatory bowel disease (IBD) as creeping fat and may explain why IBD is often associated with inflammatory skin disorders [38]. A similar mechanism of communication from the skin to the gut after cutaneous inflammation from ircAEs may play a role in the explanation of our relatively high frequency of observance of GI irAEs following ircAEs.

Considering our data, it must be clearly stated that the skin—gut axis is by no means the only pathway to GI irAE. Rather, as there are multiple proposed mechanisms directly leading to ircAE or GI irAE, we believe that communication through the skin—gut axis and perhaps the gut—skin axis can further contribute and promote irAE in the respective organs.

The timely diagnosis and management of ircAEs may prevent development of subsequent GI irAEs or diminish the severity of subsequent irAEs. Prospective trials to determine optimal approaches in irAE management are lacking; thus, guidelines rely on expert consensus. Currently, recommendations for grade 1 ircAEs include the initiation of moderate to high-potency topical corticosteroids with a continuation of ICB. For grade 2 ircAEs, systemic corticosteroids may be started with a continuation of ICB. In instances of grade 3+ ircAEs, ICB should be held until the ircAE resolves to grade 1 or 0, while systemic corticosteroids are initiated promptly. These guidelines are summarized in Table 4 with additional special considerations for specific ircAE phenotypes such as vitiligo, bullous pemphigoid, and Stevens—Johnson syndrome/toxic epidermal necrolysis (SJS/TEN). Depending on ircAE phenotype, biologics such as omalizumab (for pruritus) and rituximab (for bullous pemphigoid) may be considered [39,40,41,42,43].

GI irAEs should similarly be treated promptly when symptoms such as diarrhea arise. Table 5 summarizes management recommendations for irColitis [44]. When irColitis from ICB is diagnosed, patients should be treated with high-dose steroids. If symptoms persist or progress, and do not improve within 2–3 days, biologics such as infliximab or vedolizumab may be considered [45,46,47]. A stool transplant may be considered in refractory or recurrent cases [48]. Prompt recognition and subspeciality involvement in the management of irAEs also allows for the selection of targeted agents that may benefit multiple toxicities, such as the use of infliximab and ustekinumab in the case of overlapping skin and gut toxicities. Further studies are necessary to determine the best modalities for irAE management that do not dampen ICB efficacy [5].

The large sample size of the two cohorts is one of biggest strengths of this study. In Cohort 1, a dermatologist with expertise in ircAE management confirmed the association of dermatologic toxicities with ICB. Similarly, in Cohort 2, all GI irAEs were reviewed and confirmed by an expert in oncological gastroenterology. Additionally, suspected ircAEs were thoroughly reviewed by a dermatologist and only ircAEs that were highly likely to be associated with ICB use were included. The limitations of this study include its retrospective nature. There is also possible under-reporting of milder ircAEs and irColitis in Cohort 2 as only patients who had biopsy-confirmed irColitis were included, and among those, only the charts of patients who had a documented dermatology visit were reviewed for ircAEs.

## 5. Conclusions

In our cohorts, we observe that GI irAEs, especially irColitis, often develop after cutaneous ircAEs in the subset of patients who develop both skin and GI irAEs with ICB. As ICB is increasingly used for the treatment of melanoma and other malignancies, physicians and patients should be cognizant of the likelihood of GI irAEs developing after skin irAEs. Based on our current findings, we hypothesize that communication within the skin—gut axis may play a role in the asynchronous development of both ircAEs and GI irAEs and that future research into the pathogenesis of these irAEs is warranted.

## Figures and Tables

**Table 1:** Demographics of patients with irAEs.

Variables		Cohort 1 (*n* = 152)		Cohort 2 (*n* = 246)		*p*-Value
		*n*	%	*n*	%	
Age (mean)		64.3		62.4		*p* = 0.255
Sex	Female	59	38.8	97	39.4	*p* = 0.903
	Male	93	61.2	149	60.6	
Race	Asian	11	7.2	10	4.1	*p* = 0.150
	Black	4	2.6	2	0.8	
	Native American	2	1.3	0	0	
	White	126	82.9	221	89.8	
	Other	4	2.6	4	1.7	
	N/A	5	3.3	9	3.7	
Ethnicity	Hispanic	9	5.9	13	5.3	*p* = 0.963
	Not Hispanic	137	90.1	223	90.7	
	N/A	6	4.0	10	4.1	
Tumor Type	Breast	1	0.7	2	0.8	*p* < 0.001
	Cervical	2	1.3	2	0.8	
	CNS	3	2.0	5	2.03	
	Colorectal	5	3.3	3	1.2	
	Endometrial	10	6.6	3	1.2	
	Gastrointestinal/Hepatobiliary	9	5.9	30	12.2	
	Genitourinary	47	30.9	39	15.9	
	Head and Neck/ Endocrine	4	2.6	9	3.7	
	Hematologic	0	0.0	8	3.3	
	Lung	31	20.1	46	18.7	
	Melanoma	25	16.5	76	30.9	
	Merkel Cell Carcinoma	4	2.6	4	1.6	
	Ovarian	2	1.3	4	1.6	
	Prostate	3	2.0	14	5.7	
	Sarcoma	6	4.0	1	0.4	
ICB Type	Atezolizumab (anti-PDL1)	11	7.2	9	3.7	*p* < 0.001
	Avelumab (anti-PDL1)	8	5.3	2	0.8	
	Cemiplimab (anti-PD1)	0	0.0	1	0.4	
	Durvalumab (anti-PDL1)	7	4.6	7	2.9	
	Durvalumab (anti-PDL1)/ Tremelimumab (anti-CTLA4)	0	0.0	7	2.9	
	Ipilimumab (anti-CTLA4)	0	0.0	35	14.2	
	Ipilimumab (anti-CTLA4)/ Nivolumab (anti-PD1)	38	25.0	71	28.9	
	Nivolumab (anti-PD1)	30	19.7	35	14.2	
	Pembrolizumab (anti-PD1)	58	38.2	76	30.9	
	Tremelimumab (anti-CTLA4)	0	0.0	2	0.8	

*p*-values represent *t*-tests (age) and chi-squared tests (sex, race, ethnicity, tumor type, ICB type) between Cohort 1 and Cohort 2.

**Table 2:** Cohort 1: GI irAE characteristics in patients with ircAEs.

Variable		Developed Only ircAEs		Developed ircAEs + GI irAEs		*p*-Value
		n	%	n	%	
**All Patients with ircAE**		126	82.9	26	17.1	
ircAE Phenotype	Bullous	4	80.0	1	20.0	*p* = 0.768
	Eczema	6	100.0	0	0.0	
	Lichenoid	2	100.0	0	0.0	
	Maculopapular	26	83.9	5	16.1	
	Pruritus	64	79.0	17	21.0	
	Psoriasis	5	100.0	0	0.0	
	Urticaria	10	90.9	1	9.1	
	Vitiligo	9	81.8	2	18.2	
ircAE Grade	1	76	81.7	17	18.3	*p* = 0.106
	2	43	89.6	5	10.4	
	3	7	63.6	4	36.4	
ICB Target	Anti-PD1/PDL1	96	84.2	18	15.8	*p* = 0.456
	Anti-CTLA4 + Anti-PD1	30	79.0	8	21.1	
ICB Antibody	Atezolizumab	9	81.8	2	18.2	*p* = 0.121
	Avelumab	4	50.0	4	50.0	
	Durvalumab	7	100.0	0	0.0	
	Nivolumab	26	86.7	4	13.3	
	Pembrolizumab	50	86.2	8	13.8	
	Ipilimumab/ Nivolumab	30	79.0	8	21.1	
Best Response to ICB	Complete Response	7	77.8	2	22.2	*p* = 0.880
	Partial Response	30	85.7	5	14.3	
	POD	29	85.3	5	14.7	
	Stable	60	81.1	14	18.9	
**Patients with Both irColitis and ircAE**				*n*	%	*p*-value
GI irAE Grade	1			15	57.7	-
	2			9	34.6	
	3			2	7.7	
Relationship to ircAE	GI irAE Before ircAE			8	30.8	*p* = 0.014
	GI irAE After ircAE			18	69.2	

*p*-values represent chi-squared tests between patients who developed only ircAEs and those who developed both ircAEs + GI irAEs. Binomial probability was assessed for ircAE preceding GI irAE using the Bernoulli model.

**Table 3:** Cohort 2: ircAEs in patients with biopsy-proven irColitis.

Variable		Developed only irColitis		Developed irColitis + ircAEs		*p*-Value
		n	%	n	%	
All Patients with Biopsy-Proven Colitis		211	85.8	35	14.2	*p* = 0.010
ICB Target	Anti-PD-1/PD-L1	114	87.7	16	12.3	
	Anti-CTLA4	36	97.3	1	2.7	
	Anti-PD-1/PD-L1 + Anti-CTLA4	61	77.2	18	22.8	
ICB Antibody	Atezolizumab	9	100.0	0	0.0	*p* = 0.125
	Avelumab	1	50.0	1	50.0	
	Cemiplimab	1	100.0	0	0.0	
	Durvalumab	6	87.7	1	14.3	
	Nivolumab	32	91.4	3	8.6	
	Pembrolizumab	65	85.5	11	14.5	
	Ipilimumab	34	97.1	1	2.9	
	Tremelimumab	2	100.0	0	0.0	
	Ipilimumab/ Nivolumab	55	76.4	17	23.6	
	Tremelimumab/ Durvalumab	6	85.7	1	14.3	
**Patients with Biopsy-Proven irColitis AND ircAE**				***n***	**%**	***p*-Value**
ircAE Phenotype	Lichenoid			4	11.4	-
	Maculopapular			13	37.1	-
	Pruritus			5	14.3	-
	Psoriasis			1	2.9	-
	Urticaria			4	11.4	-
	Vitiligo			2	5.7	-
	Other			6	17.1	-
ircAE and GI irAE Relationship	ircAE Before irColitis			26	74.3	*p* = 0.0001
	irColitis Before ircAE			9	25.7	

*p*-values represent chi-squared tests between patients who developed only ircAEs and those who developed both ircAEs + GI irAEs. Binomial probability was assessed for ircAE preceding GI irAE using the Bernoulli model.

**Table 4:** Management recommendations for ircAEs.

ircAE Grade	General Recommendations	Phenotype-Specific Recommendations
1	Start moderate- to high-potency topical corticosteroids Continue ICB	-
2	Consider adding systemic corticosteroids (prednisone 0.5–1 mg/kg daily) Continue ICB	Psoriasiform rash: Consider narrow-band UVB phototherapy or apremilast Pruritus: Consider GABA analogs Bullous Pemphigoid: Hold ICB until grade 0 or 1
3+	Start systemic corticosteroids (prednisone 0.5–2 mg/kg daily) Hold ICB until grades 0–1	Maculopapular or lichenoid rash: Consider infliximab or tocilizumab Psoriasiform rash: Consider ustekinumab, guselkumab, infliximab, adalimumab, apremilast, or retinoids Pruritus: Consider GABA analogs, omalizumab, or dupilumab Bullous Pemphigoid: Consider rituximab SJS/TEN: Hospitalization

**Table 5:** Management recommendations for irColitis.

Step	General Recommendations	Specific Recommendations
1	Supportive treatment Continue ICB	Diarrhea: Loperamide, hydration, dietary modifications
2–3	Early endoscopic evaluation Administer systemic corticosteroids (prednisone 1–2 mg/kg daily) Hold ICB	Systemic symptoms (fever, tachycardia, etc.): Hospitalization Steroid-refractory cases: Consider biologics such as infliximab or vedolizumab Biologics-refractory cases: Consider stool transplant
4	Hospitalization Intravenous corticosteroids (methylprednisolone 1–2 mg/kg daily) Permanently discontinue ICB	Systemic symptoms (fever, tachycardia, etc.): Hospitalization Steroid-refractory cases: Consider biologics such as infliximab or vedolizumab Biologics-refractory cases: Consider stool transplant

## Data Availability

Fully anonymized data are available upon request.

## Supplementary Materials

The following supporting information can be downloaded at: https://www.mdpi.com/article/10.3390/cancers14122995/s1, Table S1: Search strategy Cohort 1; Table S2: Sub-cohort analysis by cancer type.
