# Supplementary material for: Contribution of the Skin–Gut Axis to Immune-Related Adverse Events with Multi-System Involvement
Source: Cancers (Basel). 2022 Jun 17;14(12):2995. doi: 10.3390/cancers14122995 (PMC9221505; doi:10.3390/cancers14122995)
Supplement: Supplementary file 1 [file cancers-14-02995-s001.zip › cancers-1730184-supplementary.pdf]

Supplementary Material: Contribution of the Skin–Gut Axis to Immune-Related Adverse Events with Multi-System Involvement

Alyce M. Kuo, Lukas Kraehenbuehl, Stephanie King, Donald Y.M. Leung, Elena Goleva, Andrea P. Moy, Mario E. Lacouture, Neil J. Shah and David M. Faleck

Table S1. Search strategy Cohort 1.

**DataLine Search Criteria for Cohort 1**
L12.0 - BULLOUS PEMPHIGOID<br>L13.9 - BULLOUS DISORDER, UNSPECIFIED<br>L20.9 - ATOPIC DERMATITIS, UNSPECIFIED<br>L27.0 - Generalized skin eruption due to drugs and medicaments taken internally<br>L29.9 - PRURITUS, UNSPECIFIED<br>L50.8 - Other urticaria<br>L50.9 - URTICARIA, UNSPECIFIED<br>L80 - Vitiligo<br>L81.6 - Other disorders of diminished melanin formation<br>L81.8 - Other specified disorders of pigmentation<br>L81.9 - DISORDER OF PIGMENTATION, UNSPECIFIED
**Criteria**
1) Patients who have had any of the following ICD-10 codes, effective the last 3 years:<br>L12.0 - BULLOUS PEMPHIGOID<br>L13.9 - BULLOUS DISORDER, UNSPECIFIED<br>L20.9 - ATOPIC DERMATITIS, UNSPECIFIED<br>L27.0 - Generalized skin eruption due to drugs and medicaments taken internally<br>L29.9 - PRURITUS, UNSPECIFIED<br>L50.8 - Other urticaria<br>L50.9 - URTICARIA, UNSPECIFIED<br>L80 - Vitiligo<br>L81.6 - Other disorders of diminished melanin formation<br>L81.8 - Other specified disorders of pigmentation<br>L81.9 - DISORDER OF PIGMENTATION, UNSPECIFIED
OR Patients with a ClinDoc in the last 3 years with the term 'rash'
2) The patients were treated with immunotherapy in the 3 months before criteria 1.: include patients on active ICB between 09/28/2017 and 09/28/2020<br>Use DataLine list or include the following:<br>CEMIPLIMAB<br>DURVALUMAB<br>IPILIMUMAB<br>ATEZOLIZUMAB<br>AVELUMAB<br>NIVOLUMAB<br>CEMIPLIMAB<br>PEMBROLIZUMAB<br>NIVOLUMAB<br>AVELUMAB<br>ATEZOLIZUMAB<br>PEMBROLIZUMAB<br>DURVALUMAB<br>MEDI0680 (AMP-514)<br>NIVOLUMAB<br>IPILIMUMAB<br>PDR001<br>SPARTALIZUMAB
3) The patients have not had a liquid primary tumor diagnosis:<br>SEER Catgegory1 in ('Lymphoma','Myeloma','Leukemia')

Table S2. Sub-cohort analysis by cancer type.

Variable	Melanoma (*n* = 25)		GU (*n* = 47)		Lung (*n* =30)		Other (*n* = 50)		*p*-value
**Cohort 1**									
	n	%	*n*	%	*n*	%	*n*	%	
ircAE Only	21	84%	36	77%	24	80%	45	90%	*P* = 0.348
ircAE + irColitis	4	16%	11	23%	6	20%	5	10%	
ircAE before irColitis	3	75%	9	82%	3	50%	3	60%	*P* = 0.547
irColitis before ircAE	1	25%	2	18%	3	50%	2	40%	
**Cohort 2**									
**Variable**	**Melanoma (*n* = 76)**		**GU (*n* = 39)**		**Lung (*n* = 46)**		**Other (*n* = 85)**		***p*-value**
	n	%	*n*	%	*n*	%	*n*	%	
Colitis Only	66	87%	28	72%	44	96%	73	86%	*P* = 0.019
ircAE + irColitis	10	13%	11	28%	2	4%	12	14%	
ircAE before irColitis	6	60%	8	73%	2	100%	10	83%	*P* = 0.515
irColitis before ircAE	4	40%	3	27%	0	0%	2	17%	

GU= genitourinary, ircAE=immune-related cutaneous adverse event, irColitis=immune-related colitis.
